# Carbohydrate Nutrition and Skill Performance in Soccer

**DOI:** 10.1007/s40279-023-01876-3

**Published:** 2023-07-08

**Authors:** Ian Rollo, Clyde Williams

**Affiliations:** 1 Gatorade Sports Science Institute, PepsiCo Life Sciences, Global R&D, Leicestershire, UK; 2 School of Sports Exercise and Health Sciences, Loughborough University, Loughborough, UK

## Abstract

In soccer, players must perform a variety of sport-specific skills usually during or immediately after running, often at sprint speed. The quality of the skill performed is likely influenced by the volume of work done in attacking and defending over the duration of the match. Even the most highly skilful players succumb to the impact of fatigue both physical and mental, which may result in underperforming skills at key moments in a match. Fitness is the platform on which skill is performed during team sport. With the onset of fatigue, tired players find it ever more difficult to successfully perform basic skills. Therefore, it is not surprising that teams spend a large proportion of their training time on fitness. While acknowledging the central role of fitness in team sport, the importance of team tactics, underpinned by spatial awareness, must not be neglected. It is well established that a high-carbohydrate diet before a match and, as a supplement during match play, helps delay the onset of fatigue. There is some evidence that players ingesting carbohydrate can maintain sport-relevant skills for the duration of exercise more successfully compared with when ingesting placebo or water. However, most of the assessments of sport-specific skills have been performed in a controlled, non-contested environment. Although these methods may be judged as not ecologically valid, they do rule out the confounding influences of competition on skill performance. The aim of this brief review is to explore whether carbohydrate ingestion, while delaying fatigue during match play, may also help retain sport soccer-specific skill performance.

## Key Points

**Table:** 

The successful execution of repeated skilled actions is a fundamental requirement for soccer performance.
Soccer players experience, to different degrees, physical and mental fatigue that have a negative impact on the performance of specific skills.
Increasing muscle and liver glycogen stores before and ingesting carbohydrate during competition delays the onset of fatigue and is conducive to maintaining the execution of soccer-specific skills.
Ingesting carbohydrate, at key times during competition, could counter negative feelings and improve concentration, helping players maintain skill execution over the duration of exercise.

## Introduction

In soccer, players must perform a variety of sport-specific skills usually during or immediately after running at various speeds. There is an obvious link between sport-specific fitness and the players’ ability to execute the relevant skill as and when it is appropriate, when defending and attacking. In all sport, skill is used as an umbrella term that includes not only physical performance of a particular skill but also the complex interaction of cognitive and technical abilities to respond to the multitude of scenarios that occur in every match. While technical skills can be taught to the point of being instinctive, the cognitive skill of being able to ‘read the game’ is one that is developed over the sporting lifespan of successful players.

Both the skill proficiency of the player and the number of specific technical actions reduce as a match progresses [1, 2]. In addition, the higher the tempo of a match, the sooner players begin to experience both physical (run, sprint, jump) and mental (concentration, decision-making) effects of fatigue, which often results in a decrease in skill performance [3, 4]. This is often to the frustration of coaches as well as spectators, who, for example, observe a misplaced shot, an ill-timed pass or a poor decision just when the team need it least. Therefore, teams dedicate a large proportion of their training time to fitness [5, 6].

Fatigue during prolonged exercise is closely associated with the depletion of the carbohydrate store (glycogen) in skeletal muscles (for full review see Ref. [7]). In a recent study of fatigue in a football match, Mohr et al. reported critically low glycogen levels in the skeletal muscles after 90 min of play and a further significant reduction following 30 min of extra time. Players ran less and performed standard skills with less accuracy than earlier in the game [8]. An early reduction in muscle and liver glycogen stores, during prolonged exercise, can be prevented by carbohydrate ingestion before and during exercise. Using this nutritional strategy, fatigue is delayed and performance sustained for longer than in the absence of this intervention [9]. In addition, several previous reviews have concluded carbohydrate ingestion also facilitates the preservation of skill performance when players are fatigued [10–12].

The aim of this paper is to discuss the most recent studies investigating the effects of carbohydrate ingestion on soccer-specific skills, and the possible role that carbohydrate ingestion plays in negating the impact that more recently reported mental fatigue has on skill performance. To inform this review article an electronic literature search was undertaken using three online databases (PubMed, Web of Science, SPORTDiscus). Searches were performed using keywords from existing relevant papers. Search terms were ‘Soccer’, ‘Football’, ‘Carbohydrate’, ‘Skill’ and ‘Performance’ phrased as appropriate. Reference lists of all studies and relevant systematic reviews were examined manually to identify relevant studies for this review.

## Skill Assessment

Skilled movements are physically complex but even more so when performed during match play because they involve an interaction between the physical and cognitive qualities necessary to achieve successful outcomes [13]. The acquisition of skills and their retention is a process that begins early in the career of soccer players. By the time they become professional players they will have achieved superior levels of soccer-specific skills, both technical and cognitive. Furthermore, hours of team training and competitions help players consolidate and extend the tactical execution of their skills. Therefore, it is not surprising that the defining characteristics of professional players are their levels of sport-specific skills in addition to their superior physical attributes [14–16].

Traditionally, a team’s and players’ level of soccer-specific skills have been assessed by the ‘experienced eye’ of coaches who know what is expected of professional soccer players. The technical components of skill fall into two large categories: closed (free kick, corners, penalties, throw-in) and open (passing, tackling, heading, goal shooting) skills [17].

In the modern game, skill performance is typically captured via team metrics from competitive matches, for example, pass completion, interceptions, shots on target, challenges won and number of interceptions [18]. An important metric is ball possession during match play. Individual players must work cohesively to create space, pass and control the ball repeatedly whilst being challenged by the opposition. Although percentage ball possession does not guarantee success, those teams with greater percentage ball possession perform more passes, touches per possession, shots, dribbles and final-third entries in comparison with teams with low percentage ball possession [19]. On-field analyses allow comparisons of how the speed and skill of the game changes, from match to match and beyond. For example, an analysis of the Men’s World Cup finals between 1966 and 2010 reported a 35% increase in the number of passes per minute of play, which was accompanied by a 15% increase in the speed of the match [20]. Nonetheless, while the team metrics obtained by ever more sophisticated match analysis technology are hugely informative, the impact of training, rehabilitation and nutritional intervention on individual players may be better understood by assessing their skills by objective assessments. Desirable as this is, it is difficult to design objective skill tests that reproduce all that goes into the successful execution of skills in competition. As a result, some studies have used isolated tests of soccer skill, for example, ball juggling [21], wall volley [22], heading [23], shooting [13, 24], passing [24–27] and dribbling [28].

Some laboratory-based studies provide controlled environments to investigate isolated skills and also attempt to simulate the physical demands of the sport. For example, the Soccer Match Simulation (SMS) protocol embeds soccer-specific skills to enhance the ecological validity of a previously validated simulated assessment of the energy demands of a soccer match [29, 30]. However, while objective tests of skill have many advantages, they are not without several limitations. Rodriguez et al. discuss the importance of playing surface on the ecological validity of soccer skills tests [27, 28]. For example, dribbling a ball at speed on a smooth floor is likely a greater challenge than executing this skill on grass. Correspondingly, the footwear worn for different surfaces may not be optimal for the skill under assessment, such as boots versus trainers when testing shooting skill. Furthermore, the use of sport-specific materials that are familiar to players, such as soccer mannequins instead of target boxes, should also be utilised [31]. Ali [17] has described the strengths and limitations of tests of soccer skill performance.

## Carbohydrate Ingestion and Skill

Fitness and skill go ‘hand-in-glove’; as players tire, they are less able to perform the relevant skills when needed [1, 2]. As mentioned earlier, there is a close association between the development of fatigue during a match and the depletion of players’ muscle glycogen stores, which becomes critical should the match go into extra time, extending play to 120 min [8]. Nutritional strategies to increase the body’s glycogen stores by providing carbohydrate before and during exercise improves endurance by delaying the depletion of this essential fuel. The effectiveness of carbohydrate ingestion applies not only to constant pace running and cycling but also to intermittent high-speed running [9], which is the common activity pattern in team sport, especially in soccer. How much carbohydrate should be consumed, and when, are questions that have led to tried and tested recommendations [5, 28, 32–37] (Table 1).

**Table 1:** Carbohydrate intake recommendations for team sport

Team sport exercise scenario	Objectives	Desired adaptation/outcome	Suggested daily carbohydrate ingestion range	Considerations
In-season training (1 game per week)	To delay physical and mental fatigue To maintain physical qualities (and improve where possible/appropriate) To keep players injury and illness free	To maintain aerobic and anaerobic fitness To at least maintain strength, power, speed To maintain lean body mass To support physical and technical performance	4–8 g/kg body mass	Range accommodates variations in loads across the micro-cycle (e.g. low load days and match day − 1 carbohydrate loading protocols) as well as individual training goals (e.g. manipulation of body composition to accommodate weight loss and fat loss or weight gain and lean mass gain). Practice competition carbohydrate ingestion regime
Match day − 1, match day and match day + 1			6–8 g/kg body mass to elevate muscle glycogen stores	Ingest 1–3 g of carbohydrate per kilogram body mass 3–4 h before a match to replenish liver glycogen stores Ingest 30 g of carbohydrate following the warm-up and during the half-time interval Ingest 1 g carbohydrate per kilogram body mass per hour with fluids after a match to start restoration of glycogen and rehydration

While adopting nutritional strategies to delay a rapid loss of the body’s glycogen stores helps players maintain their work rate during matches, the question is whether it also helps prevent a loss of skill? A simple answer would be that if players tire less readily, after implementing a carbohydrate feeding strategy, then they would be better able to execute the necessary skills in match play. Unfortunately, there are too few studies to provide a definitive answer to this question. However, one study reported that when male professional soccer players ingested either a 7% carbohydrate–electrolyte or placebo beverage before (5 ml per kilogram body mass) and every 15 min (2 ml per kilogram body mass) during a 90 min on-field soccer match and then completed the assessment of four skills, dribbling speed, coordination, precision and power, there was a significantly improved retention of dribbling speed and precision following carbohydrate ingestion [38].

In an innovative study on the impact of carbohydrate ingestion on skill, tests were undertaken on players’ dominant and non-dominant limbs. Using a soccer-specific protocol, higher passing scores were achieved by both dominant and non-dominant feet following the ingestion of carbohydrate (30 g, before and at half time, compared with placebo whilst drinking water ad libitum) [27]. This effect was evident from 60 min onwards. Importantly, improved performance was attained without loss of passing speed, which was better maintained in the non-dominant foot with carbohydrate ingestion. This observation is of interest because it is consistent with other studies in sports such as tennis, where non-dominant or weaker side (backhand) shots respond positively to carbohydrate ingestion, especially when fatigued [39]. The assessment of complex skilled actions on the non-dominant side may require greater activation of the central nervous system (CNS) and therefore be more susceptible to fatigue [27]. Furthermore non-dominant skilled actions may be more likely influenced by the arousal level of the player [40]. Thus, the performance of players’ non-dominant sides appears to have a greater sensitivity to carbohydrate ingestion [27], even though the ‘non-dominant’ side is likely to be inferior in performing skills.

## Carbohydrate Ingestion and Mental Fatigue

The physiology of fatigue has been extensively studied [41]. A recent model of motor or cognitive task induced fatigue proposes that no single factor is responsible for declines in skill performance. Instead, fatigue is considered a psychophysiological condition. Motor fatigue and perceived fatigue are interdependent but hinge on various determinants and depend on modulating factors such as age, sex and specific skill characteristics [42]. Mental fatigue is defined as a psychobiological state that arises during prolonged demanding cognitive activity and results in an acute feeling of tiredness and/or a decreased cognitive ability as well as mood changes [43, 44]. Mental fatigue can reduce physical capacity, assessed through reduced time to exhaustion and elevated rating of perceived exertion (RPE) [45], and has been shown to fluctuate throughout a competitive season [46]. To highlight this point, mental fatigue has been found, in one review, to have a negative influence on 37% of soccer-specific skills (*n* = 92) [43].

Mental fatigue has been recognised as a key consideration in team sport, due to the associated negative impact on physical, technical, decision-making and tactical performance [47]. Contributing factors to mental fatigue in team sport environments include but are not limited to prolonged cognitive demands, team meetings, travel and the inability to ‘switch off’ [48, 49].

Of note is the approach taken in laboratory studies which use the repeated execution of inherent sport-specific skills to induce mental fatigue [50]. Thus, tracking skill execution may also be important because it might reflect the presence of both mental and physical fatigue. Correspondingly, monitoring mental fatigue has been recommended in team sport to provide an overall picture of how players are coping with the demands of training and competition [51]. Therefore, strategies are used to help avoid mental fatigue, for example, displacement activities, such as changes in training routines, environment and, of course, adequate rest and recovery. Increasing dietary carbohydrate while improving exercise capacity both in training and in competition may also be a mood-changing countermeasure to mental fatigue [52, 53]. If players are feeling good rather than bad (pleasure–displeasure) and energized (i.e. in an activated state) before and during matches, then it is more likely that they will perform better [40, 54]. For example, Backhouse et al. have shown that the ingestion of carbohydrate elevated perceived activation during the final 30 min of 120-min of intermittent running exercise [55] and also attenuated the decline in pleasure–displeasure during a 120-min bout of cycling [56]. Administering both a Feeling Scale (FS) and an RPE scale allows a measure of not only ‘what’ (RPE) but also ‘how’ (FS) a person feels [57] but is rarely administered during skill intervention studies or applied settings.

A recent review identified mouth rinsing and expectorating a carbohydrate beverage as a potential acute countermeasure to mental fatigue [58]. The recognition of carbohydrate in the mouth, when administered immediately after a mentally fatiguing task, was linked to increased excitability of corticomotor pathways [59, 60]. Furthermore, there appears to be a direct link between improvements in task-specific activity and activation within the primary sensorimotor cortex in response to oral carbohydrate signalling [61]. These results contribute to a possible explanation for improved high-intensity intermittent running performance in response to mouth rinsing with a 10% carbohydrate beverage [62, 63]. Although not all studies report this effect [64], central activation mediated by the ingestion of carbohydrate may contribute to the better retention of sprint and technical performance observed early in exercise or in the absence of hypoglycaemia [27, 28, 65]. While mouth rinsing with a carbohydrate beverage has been shown to benefit complex whole-body skilled actions in fencers, compared with taste-matched placebos [66], the impact on soccer skill performance is yet to be investigated. Furthermore, it is also important to note that mouth rinsing with non-sweet carbohydrate activates the reward centres of the brain and so may contribute to the ‘feel good’ sensation that may counter mental fatigue [67]. Nevertheless, these findings should be considered as an additional benefit to carbohydrate ingestion, during or after exercise, when substrate delivery and replenishment of glycogen stores are the respective priorities [68–70].

These responses to carbohydrate ingestion may not be surprising bearing in mind that glucose is the main fuel for the brain and CNS [71]. For optimum functioning of the brain and CNS, glucose homeostasis must be maintained even during a wide range of conditions. Should blood glucose fall to hypoglycaemic levels, then the neural drive to skeletal muscles will be compromised; however, it is restored following the ingestion of carbohydrate [72]. During exercise, the rate of glucose release from the liver into the blood increases to match the glucose uptake by contracting muscle [73]. In most team sport, blood glucose concentrations are well maintained over the duration of competition (80–90 min) and extra time (120 min in soccer) in well-fed individuals [74]. Nevertheless, carbohydrate ingestion at the onset of exercise is an effective strategy not only to top up muscle glycogen stores but also because it temporarily inhibits hepatic glucose release in a dose-dependent manner, and so conserves liver glycogen stores [75, 76]. Carbohydrate ingestion, as a means of preserving the finite store of liver glycogen, will maintain blood glucose concentrations and performance late in exercise. This strategy is particularly beneficial when matches extend to extra time [8, 77]. Of interest is the observation that elevated blood glucose concentrations are associated with improved skill performance in comparison with euglycaemia [27, 28, 65, 78]. An immediate explanation for this observation is not apparent other than that glucose is a fuel for the brain [79, 80]. However, the brain is sensitive to changes in blood glucose, and the rate of change may act to monitor the availability of whole-body carbohydrate stores.

## Conclusion

**Fig. 1:**
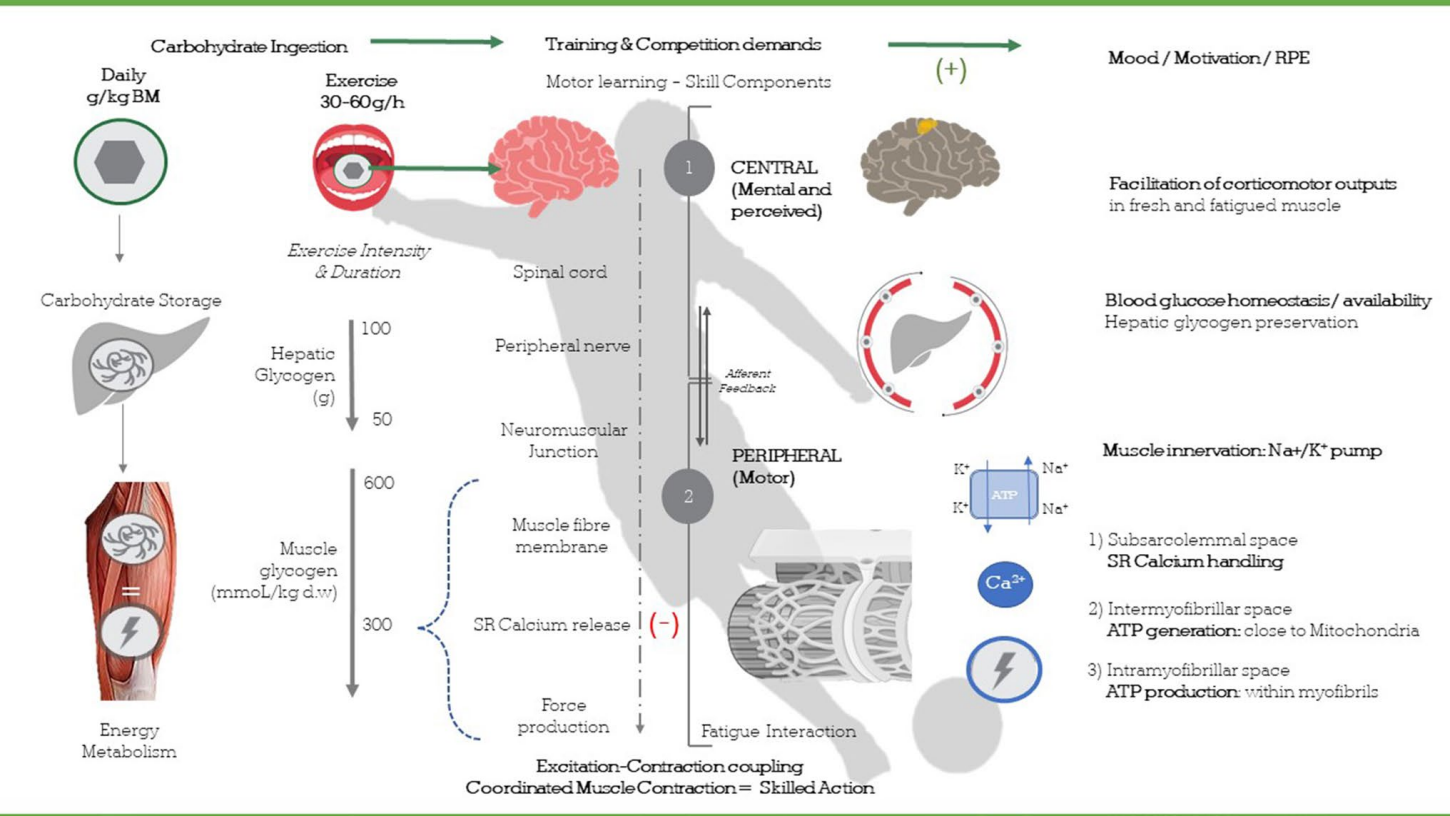
Translating thoughts into skilled actions. The electro-chemical chain of events between the brain and skeletal muscles, and how carbohydrate ingestion may impact skill performance. *BM* body mass, *SR* sarcoplasmic reticulum, *Ca*^*2+*^ calcium, *Na*^*+*^*/K*^*+*^ sodium–potassium pump, *ATP* adenosine triphosphate. ‘+’ = positive influence upon, ‘−’ = negative influence upon. Mood, motivation, RPE [52, 55, 58], facilitation of corticomotor outputs [60, 61], blood glucose availability, hepatic glycogen preservation [75, 76, 81, 82], muscle innervation: SR calcium handling [83], ATP generation [83–85]

Participants in team sport experience, to different degrees, physical and mental fatigue that have a negative impact on the performance of sport-specific skills. The complex series of events between brain and skeletal muscle that interact to minimise the impact of physical and mental fatigue on the performance of skills during competition, following carbohydrate feeding, is summarised in Fig. 1. Nutritional strategies that increase muscle and liver glycogen stores prior to competition and provide carbohydrate during competition maintain work rate by delaying the onset of fatigue. This effect of carbohydrate ingestion is, in itself, conducive to maintaining the execution of sport-specific skill. Furthermore, ingesting carbohydrate, at key times during competition, could counter negative feelings and improve concentration, thereby helping players maintain skill execution over the duration of exercise.
